# Alterations of cerebral perfusion in asymptomatic internal carotid artery steno-occlusive disease

**DOI:** 10.1038/s41598-017-02094-4

**Published:** 2017-05-12

**Authors:** Ya-Fang Chen, Sung-Chun Tang, Wen-Chau Wu, Hsien-Li Kao, Yen-Shu Kuo, Shun-Chung Yang

**Affiliations:** 1 Graduate Institute of Oncology, National Taiwan University, No. 1, Sec. 1, Ren-Ai Road, Taipei, 100 Taiwan; 2 Department of Medical Imaging, National Taiwan University Hospital, No. 7, Zhong-Shan S. Road, Taipei, 100 Taiwan; 3 Department of Neurology, National Taiwan University Hospital, No. 7, Zhong-Shan S. Road, Taipei, 100 Taiwan; 4 Department of Internal Medicine, National Taiwan University Hospital, No. 7, Zhong-Shan S. Road, Taipei, 100 Taiwan; 5 Graduate Institute of Biomedical Electronics and Bioinformatics, National Taiwan University, No. 1, Sec. 1, Roosevelt Road, Taipei, 106 Taiwan; 6 Graduate Institute of Clinical Medicine, National Taiwan University, No.1, Sec. 1, Ren-Ai Road, Taipei, 100 Taiwan; 7 Department of Radiology, Cathay General Hospital, No. 280, Sec. 4, Ren-Ai Road, Taipei, 106 Taiwan

## Abstract

Patients with asymptomatic occlusion in the internal carotid arteries (ICA) have been shown to have a better preserved hemodynamic status of the brain as compared to patients with symptoms. This study was aimed to explore the cerebral perfusion alterations in asymptomatic patients using multi-parametric arterial spin-labeling (ASL) magnetic resonance (MR) imaging. Forty-two patients diagnosed with asymptomatic ICA stenosis/occlusion were prospectively included and divided into high-grade (ultrasonographic stenosis ≥70%, N = 20) and low-grade groups (N = 22). On a 3-Tesla clinical MR scanner, pseudo-continuous ASL was performed to measure cerebral blood flow CBF, arterial transit time ATT, and flow territory. Fisher’s exact test indicates that the high-grade group has higher frequency in asymmetric ATT (p < 10^−3^) and asymmetric flow territory (p < 10^−3^) as compared to the low-grade group. The between-group difference in CBF asymmetry is marginal (p = 0.062). Logistic regression further reveals that hemispherical asymmetry in ATT and flow territory is associated with the existence of high-grade ICA stenosis (odds ratio = 12 and 21, respectively), whereas hemispherical asymmetry in CBF is not. Our data suggest that ATT and flow territory may be better predictors of asymptomatic high-grade ICA stenosis diagnosed by carotid ultrasonography than CBF.

## Introduction

It has been shown previously that patients with asymptomatic occlusion in internal carotid arteries (ICA) have a better preserved hemodynamic status of the brain as compared with symptomatic patients^1^. The preservation can be achieved by collateral circulation, usually accompanied by prolonged arterial transit time (ATT), to provide an adequate amount of perfusion (microvascular blood flow). Although the presence of a primary collateral pathway is thought to protect against cerebral ischemia^2^, collateral flow may be a herald of subsequent stroke.

ICA steno-occlusive disease has been diagnosed mostly by ultrasonography and angiography, with which flow pattern and anatomy of arteries can be quantitatively assessed. Alterations in flow velocity and/or the existence of narrowed lumen have been found to associate with transient ischemic attack and stroke^3, 4^, but hemodynamic change/compensation in the microvascular regime cannot be ruled out before symptoms. Several clinical imaging methods are available for cerebral perfusion assessment. The amount of perfusion (also referred to as cerebral blood flow, CBF) can be quantified by dynamic contrast-enhanced computed tomography^5, 6^, H_2_^15^O positron emission tomography^7^, and dynamic susceptibility-contrast magnetic resonance (MR) imaging^8, 9^. The signal evolutions of these methods can also be used to estimate transit times (e.g., mean transit time^5^ and time-to-maximum of the residue function)^10^. Digital subtraction angiography (DSA) allows flow territory depiction but requires catheter maneuver and exposure to dye and x-ray.

Arterial spin labeling (ASL)^11, 12^ is an MR imaging technique for perfusion measurement with no need of exogenous contrast material and ionizing radiation. ASL imaging magnetically labels the protons in arterial blood and then uses them as an endogenous tracer. Briefly, radiofrequency (RF) pulses are applied to invert or saturate the longitudinal magnetization of arterial blood and after a delay time to allow the labels to reach capillaries, a “label image” is acquired. A “control image” with no net perturbation to the longitudinal magnetization of arterial blood is usually acquired to control for magnetization transfer effect. CBF is then calculated from the signal difference between the label image and the control image. Arterial transit time (ATT) can be estimated by performing ASL imaging at multiple delay times^13, 14^. Territorial ASL^15, 16^ allows depiction of flow territories by using RF pulses tailored to label a single or a subset of arteries.

It remains unclear whether or to what extent cerebral perfusion alterations exist in asymptomatic ICA steno-occlusive disease. In this study, we presented the first attempt to investigate cerebral perfusion alterations in the disease by simultaneously assessing CBF, ATT, and flow territory, derived from ASL MR imaging.

## Materials and Methods

### Subjects

This study was approved by the Institutional Review Board at National Taiwan University Hospital and conducted in compliance with the Helsinki Declaration (2000). From July 2013 to September 2015, we prospectively recruited 74 patients in whom carotid ultrasonography indicated stenosis in the ICA but adequate flow amount in the vertebral arteries (VA). All patients underwent MR imaging and neurological assessment. The mean time interval between the three types of examinations was 1.2 months (from 1 week to 4 months). Thirty-two of them were excluded due to having transient-ischemic-attack-related symptoms within the past 6 months (symptomatic; N = 20) or unconfirmed symptom status (N = 12). At last, 42 patients were confirmed to be asymptomatic and included in the following analyses. Because the clinical decision of initiating carotid artery revascularization is based on the detection of a 70% or greater stenosis^17^, these patients were further divided into two groups according to ultrasonographic diagnosis: high-grade group for ≥70% stenosis and low-grade group for <70% stenosis. None of them had high-grade stenoses in both ICAs. Also included were twelve non-diseased control subjects in whom carotid ultrasonography indicated normal ICAs and adequate flow amount in VAs. Grouping and demographics are summarized in Table 1. All participants provided individual written informed consent before the initiation of any study-specific examinations. No adverse events were recorded.

**Table 1:** Subject grouping and demographics.

	High-grade group	Low-grade group	Control group	
N	20	22	12	
Age (years)	67 ± 10	59 ± 14	57 ± 13	p = 0.087
	(range = 52–82)	(range = 32–79)	(range = 35–72)	
Sex (female/male)	8/12	8/14	6/6	p = 0.726
Degree of stenosis at the more severe ICA	>70% to occlusion	20–60%	—	—
Degree of stenosis at the contralateral ICA	Normal to 60%	Normal to 20%	—	—

Age is expressed as mean ± standard deviation. Between group comparison: Kruskal-Wallis test for Age and Fisher’s exact test for Sex.

### MR Imaging

**Figure 1:**
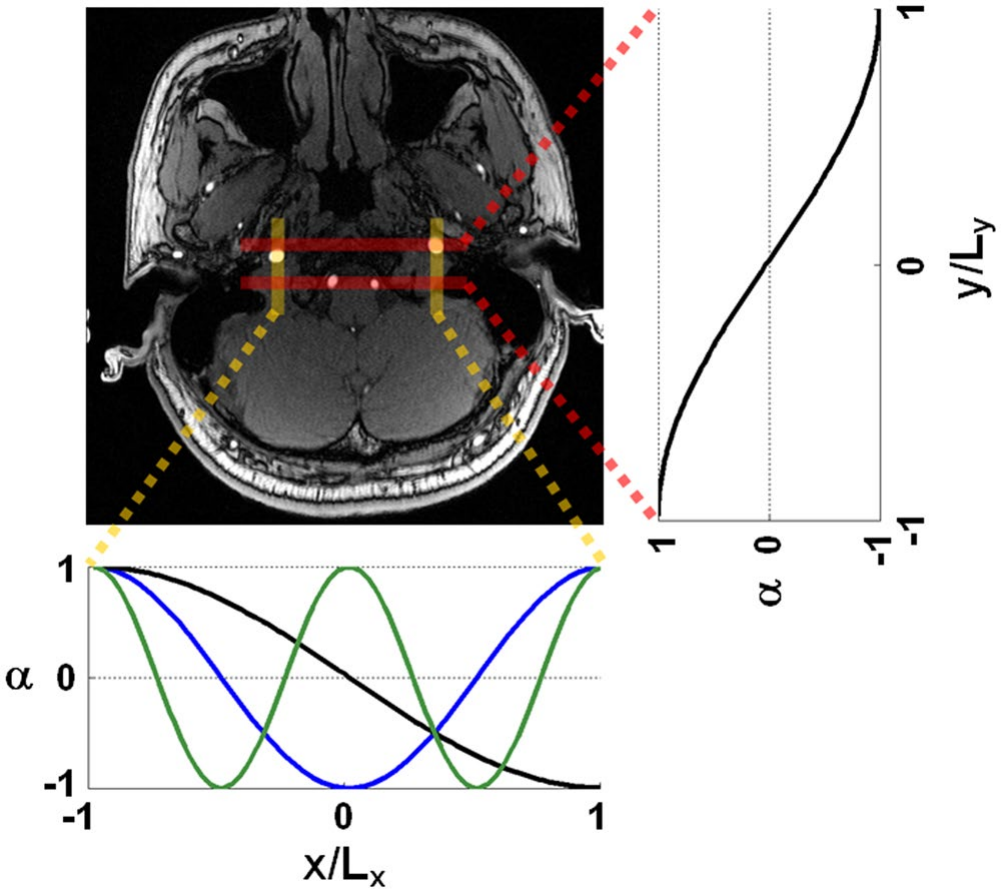
Labeling layout. Labeling was planned by referring to a time-of-flight image where internal carotid arteries and vertebral arteries were most identifiable and most perpendicular to the labeling plane. Labeling efficiency (α) was approximately a sinusoidal function of location within a spatial period of encoding (denoted by L_x_ in the left-right direction and L_y_ in the anterior-posterior direction, respectively). In this study, 4 steps were played out as shown by sinusoidal curves (1 step in the anterior-posterior direction and 3 harmonic steps in the left-right direction).

All MR imaging was performed on a 3-Tesla clinical system (Tim Trio, Siemens) using the body coil to transmit RF pulses and phased-array head (12 channels) and neck (4 channels) coils to receive signals. Imaging protocol included fluid-attenuated inversion recovery (FLAIR), T2-weighted turbo spin echo, T1-weighted imaging (turbo spin echo and three-dimensional magnetization-prepared rapid gradient echo), time-of-flight imaging, contrast-enhanced angiography, and post-contrast T1-weighted imaging. For ASL imaging, pseudo-continuous labeling^18, 19^ was employed, followed by single-shot gradient-echo echo-planar readout (TE = 13 ms, in-plane matrix = 64 × 64, 16 slices, slice thickness = 5 mm, inter-slice gap = 1 mm). For CBF measurement, TR = 5 s, labeling duration (τ) = 2 s, post-labeling delay (PLD) = 2000 ms, and 30 repetitions were obtained (i.e., 15 pairs of label and control). For ATT measurement, TR = 4 s, τ = 1 s, and PLD was varied in separate scans (200, 500, 800, 1100, 1400, 1700, and 2000 ms; 10 repetitions for each PLD). For flow territory mapping, TR = 5 s, τ = 2 s, PLD = 2000 ms, and 4 steps of harmonic encoding^20^ (8 repetitions for each step) as shown in Fig. 1. Briefly, vessel-encoding gradients were adjusted to create labeling profiles of spatial frequencies that were integer times the fundamental spatial frequency. If one of the ICAs was not identifiable, its location was replaced by the left-right symmetric location of the contralateral ICA. At last, reference scans were performed for labeling efficiency (α) calibration and receiver sensitivity correction^21^.

### Image Processing and Analysis

Image processing and analysis were performed using custom-designed programs in MATLAB (The MathWorks Inc., Natick, MA, USA). ASL images were realigned to remove head motion during the scan using the Statistical Parametric Mapping software (http://www.fil.ion.ucl.ac.uk/spm/). Spatial variation of receiver sensitivity was corrected for using a sensitivity map generated by dividing the reference image acquired with the phased array by the reference image acquired with the body coil^21^. For each series, control and label images were pair-wise subtracted and then averaged to generate the difference signal Δ*M*. CBF was calculated by

1 $${\rm{CBF}}=\frac{{\rm{\Delta }}M}{2{\rm{\alpha }}{M}_{{\rm{0A}}}{T}_{{\rm{1A}}}[\exp (-\frac{PLD}{{T}_{{\rm{1A}}}})-\exp (-\frac{PLD+\tau }{{T}_{{\rm{1A}}}})]}$$

where *M*_0A_ and *T*_1A_ are the fully-relaxed longitudinal magnetization and longitudinal relaxation time constant of arterial blood, respectively. *M*_0A_ was estimated by using ventricular cerebrospinal fluid as an internal reference and a scaling factor of 0.85^21^. *T*_1A_ was assumed to be 1660 ms^22, 23^. To estimate ATT, multi-PLD data was fitted with the model described in ref. 24 subject to the following criteria: lower/upper limits = 0/2500 ms and R-square ≥0.9. Flow territory was calculated using independent component analysis and fuzzy clustering as described in ref. 20, which yielded for each voxel membership levels of flow territories (left ICA, right ICA, and VA). Existence of asymmetric changes in CBF, ATT, and flow territory were assessed independently by two raters (with 15 years of experience in neuroradiology and 10 years of experience in brain image analysis, respectively) with difference resolved by consensus.

### Statistical Analysis

Fisher’s exact test was performed to compare between high-grade and low-grade groups the frequency of perfusion asymmetry (for CBF, ATT, and flow territory, separately). Binary logistic regression was then performed by including the three parameters as dichotomous predictors to compare their relations with the severity of stenosis. The significance level was set to 0.05.

## Results

**Figure 2:**
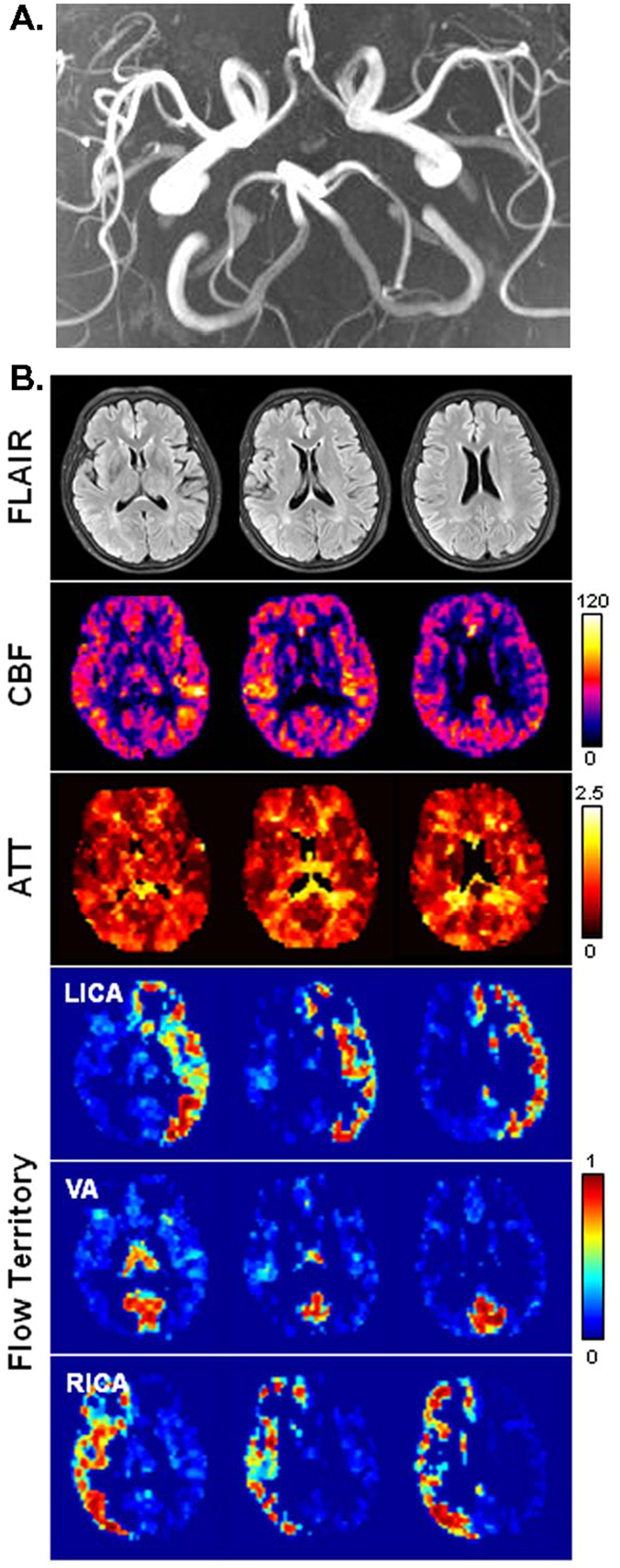
MR images obtained from a 65-year-old female patient with asymptomatic ICA steno-occlusive disease (10–30% stenosis in the left ICA and no stenosis in the right ICA). (**A**) Time-of-flight angiography. Projection view from below. (**B**) From top to bottom rows (3 slices are shown in radiological convention): FLAIR images, CBF maps (in units of ml-blood/100 ml-tissue/min), ATT maps (in units of sec), and flow territory maps. Flow territory maps show the membership levels generated by fuzzy clustering, which range from 0 to 1.

Figure 2A,B shows a representative low-grade patient who had a mild stenosis (10–30%) in the left ICA and no stenosis in the right ICA as revealed by carotid ultrasonography. No asymmetry is observed in CBF and ATT maps. Flow territories of the left ICA and the right ICA are both obtainable, appearing largely symmetrical to each other. The flow territory of vertebral arteries (VA) can also be identified to include the occipital area and thalami.

**Figure 3:**
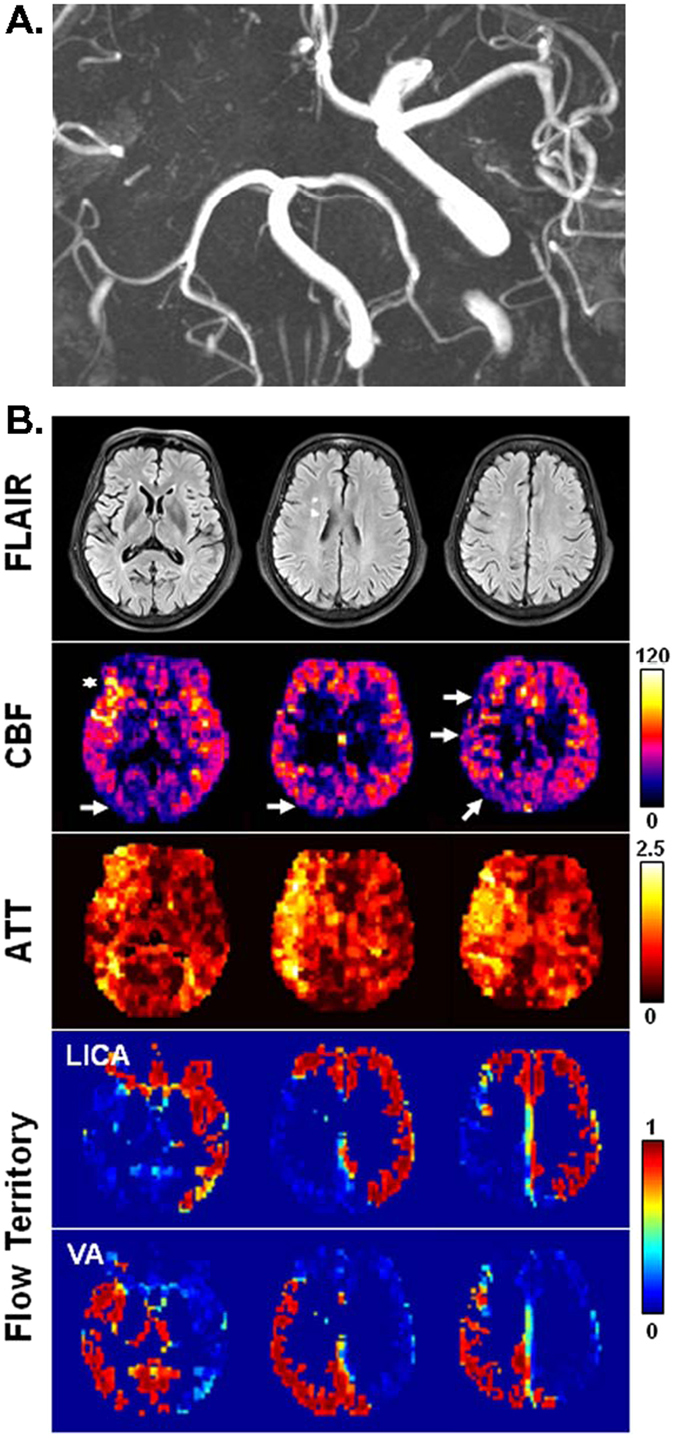
MR images obtained from a 60-year-old female patient with asymptomatic ICA steno-occlusive disease (occlusion in the right ICA and 30–50% stenosis in the left ICA). (**A**) Time-of-flight angiography. Projection view from below. (**B**) From top to bottom rows (3 slices are shown in radiological convention): FLAIR images, CBF maps (in units of ml-blood/100 ml-tissue/min), ATT maps (in units of sec), and flow territory maps. Flow territory maps show the membership levels generated by fuzzy clustering, which range from 0 to 1. No contribution from right ICA was extractable. In FLAIR images, there are two small foci of white matter hyperintensity in right frontal white matter which might represent chronic ischemic white matter lesions.

Figure 3A,B shows a high-grade patient in whom the right ICA was occluded and the left ICA had a mild stenosis (30–50%). As expected, no flow territory of right ICA was extractable. The left ICA supplies the left cerebrum including the MCA (middle cerebral artery) and ACA (anterior cerebral artery) territories and also the right anterior and medial frontal lobe which corresponds to a larger than normal right ACA territory. In addition to the occipital areas, the VA flow also supports the right posterior frontal and temporo-parietal area (the majority of the right MCA territory). Increased ATT is found in the right temporal area and parietal area. CBF is slightly decreased in the right parieto-occipital area (indicated by arrows) and intravascular hyperintensity is noted in the right MCA (indicated by asterisk), suggesting prolonged transit time.

**Figure 4:**
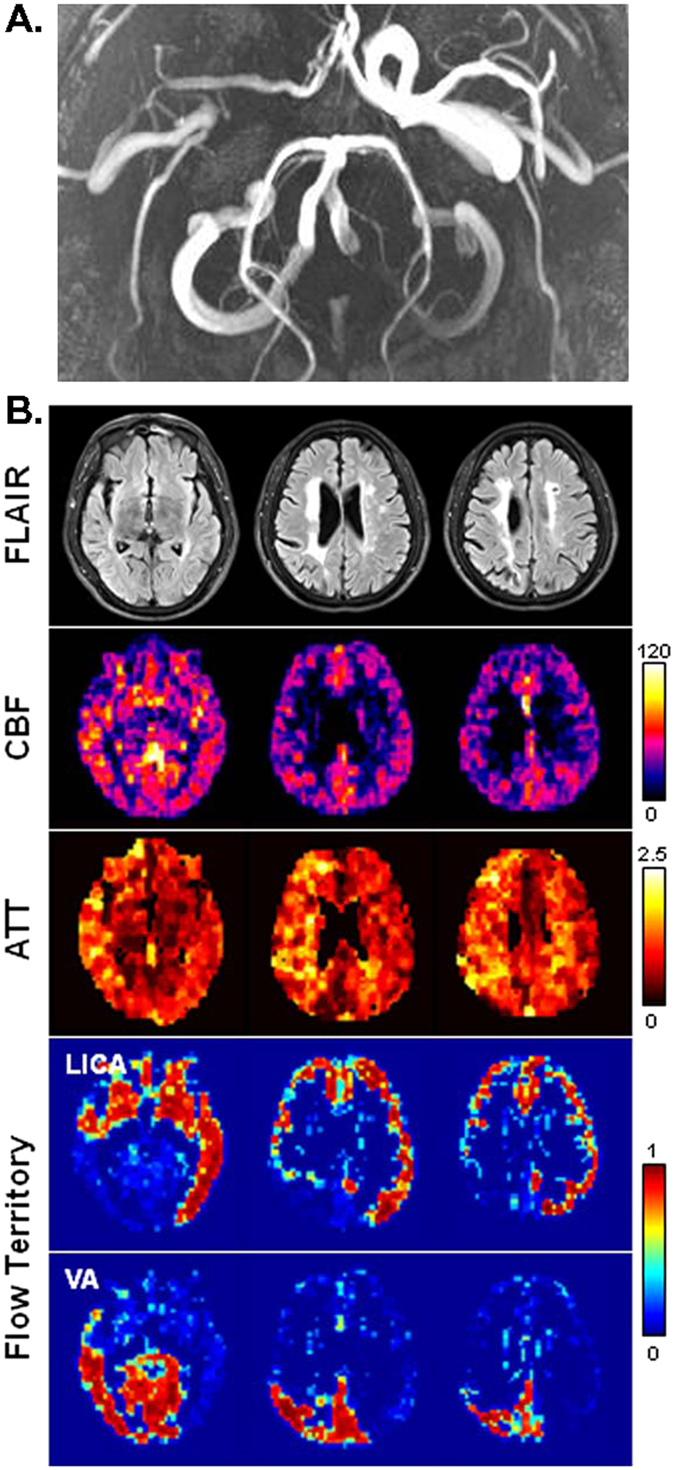
MR images obtained from a 57-year-old male patient with asymptomatic ICA steno-occlusive disease (>95% stenosis in the right ICA and 10–30% stenosis in the left ICA). (**A**) Time-of-flight angiography. Projection view from below. (**B**) From top to bottom rows (3 slices are shown in radiological convention): FLAIR images, CBF maps (in units of ml-blood/100 ml-tissue/min), ATT maps (in units of sec), and flow territory maps. Flow territory maps show the membership levels generated by fuzzy clustering, which range from 0 to 1. No contribution from right ICA was extractable. In FLAIR images, there are bilateral confluent periventricular white matter hyperintensities which are larger on the right side.

Figure 4A,B shows another high-grade patient with a severe stenosis (>95%) in the right ICA and a mild stenosis (10–30%) in the left ICA. ATT is noticeably increased in the right hemisphere, whereas CBF remains largely symmetrical. The flow territory of right ICA is not detectable, presumably due to extremely low flow. The left ICA supplies the left cerebrum including the MCA and ACA territories and also the right frontal lobe and anterior part of right temporal lobe, which correspond to the right ACA and anterior portion of the right MCA territory. The VA flow supports the occipital area and right temporo-parietal area (the posterior portion of right MCA territory).

Fisher’s exact test indicates that the high-grade group tends to have a higher frequency in asymmetric ATT (p < 10^−3^) and asymmetric flow territory (p < 10^−3^) as compared to the low-grade group (Table 2). The between-group difference in CBF asymmetry is marginal (p = 0.062). Logistic regression further reveals that hemispherical asymmetry in ATT or flow territory is associated with the existence of high-grade ICA stenosis (odds ratio = 11.8 and 21.2, respectively), whereas hemispherical asymmetry in CBF is not (Table 3). As far as the low-grade group and the control group are concerned, no between-group difference was found in the frequency of asymmetric ATT, flow territory, and CBF (Table 2).

**Table 2:** Frequency of perfusion asymmetry in high-grade group (N = 20), low-grade group (N = 22), and control group (N = 12).

	High-grade group	Low-grade group	Control group	Fisher’s exact	
				High-grade vs. Low-grade	Low-grade vs. Control
ATT	17	3	0	p < 10^−3^	p = 0.537
Flow territory	17	2	2	p < 10^−3^	p = 0.602
CBF	7	2	0	p = 0.062	p = 0.529

**Table 3:** Binary logistic regression analysis of 42 asymptomatic ICA steno-occlusive patients.

	B	Wald’s χ^2^	p	Exp(B)
Constant	−2.730	—	—	—
ATT	2.469	5.436	0.020	11.808
Flow territory	3.053	7.954	0.005	21.178
CBF	0.638	0.146	0.702	1.893

Three dichotomous predictors were included (ATT, flow territory, and CBF): 1 = asymmetry and 0 = no discernable asymmetry. Hosmer-Lemeshow test yielded a χ^2^(3) of 0.792 and was insignificant (p = 0.851), suggesting that the model fit the data well.

## Discussion

Although CBF has been used to assess ischemic stroke and help treatment formation (e.g., by demarcating penumbra), our data indicate that CBF provides marginal predictive value for asymptomatic low-grade ICA stenosis, presumably because the overall blood flow is relatively well-preserved in asymptomatic patients. However, the preservation of CBF may involve hemodynamic changes as manifested in ATT increase and/or altered route of blood supply. As compared to low-grade patients, high-grade patients are more likely to have altered flow territory (odds ratio = 21.2) and prolonged ATT (odds ratio = 11.8). ATT and flow territory may therefore serve as early imaging markers for the status of ICA stenosis in asymptomatic patients.

By using a different territorial ASL technique, a recent study^25^ investigated the flow territory change in 23 patients who had symptomatic carotid artery occlusion. In the 18 patients with unilateral ICA occlusion, the contralateral ICA was found to supply a large part of the ACA flow territory ipsilateral to the side of the ICA occlusion while the flow territory of the vertebrobasilar arteries substantially extended into the MCA flow territory on the side of the ICA occlusion. Our data has revealed similar flow territory changes in patients with asymptomatic unilateral high-grade stenosis, suggesting that flow territory change precedes symptoms. Although based on our patient number, we were unable to determine at what degree of stenosis would flow territory start to change or whether the change is moderated by the degree of stenosis on the contralateral side. Further investigation including a larger number of patients that are more evenly distributed to different degrees of stenosis is required to answer this question.

ATT and flow territory maps allow detection of hemodynamic change before CBF deficit occurs, which may help predicting the area prone to border zone infarct. The combination of CBF/ATT/flow territory may also help understanding the equivocal association between asymptomatic ICA stenosis and cognitive decline reported previously^26, 27^. For example, the area with prolonged ATT and located at the border between altered flow territories may be subject to unstable perfusion and therefore silent stroke. One can compare the type of cognitive impairment (e.g., its functional area in the brain) with multi-parametric maps to confirm or rule out the effect of carotid stenosis.

**Figure 5:**
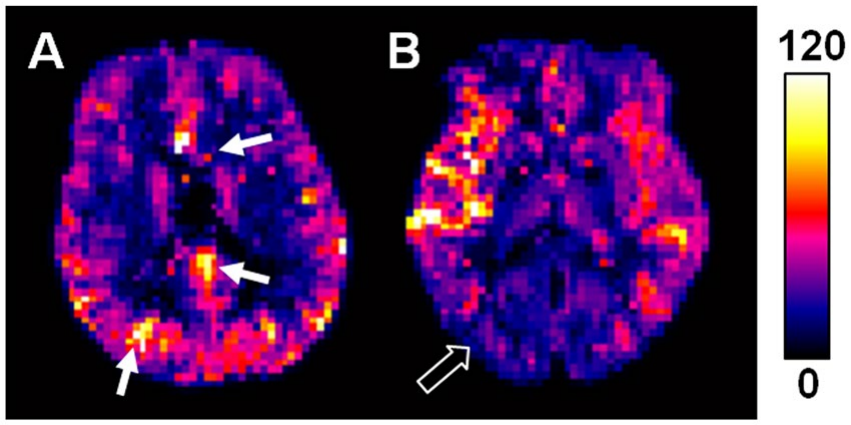
Representative CBF maps (in units of ml-blood/100 ml-tissue/min) derived from multi-PLD data for (**A**) the rightmost slice in Fig. 2 and (**B**) the leftmost slice in Fig. 3. Arrows indicate isolated hyperintensities. The open arrow indicates regional hypointensity as compared to single-PLD-derived value.

It is noteworthy that ASL imaging is not the only technique available in clinical settings for assessment of ATT, CBF, and flow territory. However, ASL imaging is currently the only technique that can measure all of the three perfusion parameters (i.e., one-stop examination) and does not require contrast administration and ionizing radiation exposure. However, ASL imaging has inherently low signal-to-noise ratio as compared to its contrast-material-based counterparts. Therefore, averages are usually required to obtain adequate signal-to-noise ratio, which leads to a relatively long scan time. In this study, it took about 13 minutes to complete ATT, CBF, and flow territory measurement (with dummy scans and calibration scans included). Although longer than contrast-enhanced MR imaging and computed tomography (usually ~2 min), it is still considerably time efficient if one considers the preparation time for DSA procedure. Also note that the scan time could be further reduced by deriving CBF and ATT from a single multi-PLD scan^28^. As shown in Fig. 5, multi-PLD-derived CBF maps are largely comparable with single-PLD-derived results (Figs 2 and 3) except for slightly more isolated hyperintensities and lower values in areas with prolonged ATT. The discrepancies could depend on signal-to-noise ratio as well as the number/interval/range of PLD, which is beyond the scope of the present study. Numerical simulation indicates that ATT is less susceptible to noise than CBF as derived from multi-PLD data (Table 4). We therefore chose to report ATT based on multi-PLD data and CBF based on single-PLD data, although using the multi-PLD-derived CBF did not change our dichotomous scoring results.

**Table 4:** Coefficient of variation of multi-PLD-derived CBF and ATT as predicted by computer simulation.

SNR_ΔM_	ATT = 900 ms		ATT = 1200 ms		ATT = 1800 ms	
	CBF	ATT	CBF	ATT	CBF	ATT
5	10.0%	9.5%	12.9%	8.0%	30.0%	10.9%
10	5.0%	4.5%	6.4%	4.0%	12.4%	4.3%
50	1.1%	1.0%	1.3%	0.8%	2.5%	0.8%

Based on the previously reported model^24^, PCASL signal (ΔM) was generated for each combination of signal-to-noise ratio (SNR_ΔM_ = 5, 10, and 50, representing low SNR, average SNR, and almost noise-free conditions, respectively) and ATT (900, 1200, and 1800 ms, approximately covering the range previously reported). For each combination, 1000 multi-PLD datasets were generated and yielded 1000 estimates of CBF and ATT, from which coefficient of variation was calculated to assess measurement variability.

Note that hemispherical asymmetry of ATT and flow territory was not found in all of our high-grade patients. This could be due to sensitivity limitation of the measurements and/or variations in individual vasculature or cerebrovascular reserve. Multi-parametric measurement is thus of clinical importance by providing parameters complementary to one another physiologically (different hemodynamic aspects) and technically (different measurement sensitivity). Further, the association between these parameters may change during disease development and progression. For example, ATT and flow territory may provide early detection and progression assessment of ICA stenosis but are probably not sufficient to determine the presence of CBF deficit. In spite of its marginal predictive value for high-grade asymptomatic ICA stenosis, CBF should play a critical role in symptomatic patients^12, 29^ considering that ATT and flow territory are altered with the ultimate aim of CBF preservation. Also note that our study was performed at the resting state. In some asymptomatic patients, challenges (e.g., using acetazolamide)^6^ may induce observable asymmetry in CBF (and/or changes in ATT and flow territory as compared to the resting state), which may provide additional information in regard to cerebrovascular reserve.

This study has a few limitations. First, no longitudinal data was presented. Second, symptomatic patients were not included for comparison. According to published guidelines^30, 31^, prompt surgical treatment is generally recommended in symptomatic patients, whereas controversies remain in the management for asymptomatic patients (medical therapy versus intensive intervention). From this perspective, exploration of asymptomatic patients alone should provide unique clinical implication considering that in these patients perfusion deficit/compensation tends to be subtle and multi-parametric ASL may help treatment formation. Third, baseline characteristics such as leucoencephalopathy and age-related hemodynamic change were not entirely controlled in our subjects. Thus, we cannot rule out the possibility that the observed asymmetry was secondary even though there was no statistical age difference between groups. This issue warrants further investigation but is beyond the scope of this study. Fourth, the asymmetry of CBF, ATT, and flow territory was assessed by dichotomous scoring and consensus of two raters. Although commonly employed in clinical radiology, the method might be hindered from further sophisticated analysis due to potential subjectivity. An alternative worth further investigation is assessing the asymmetry in terms of similarity (e.g., Dice’s coefficient) in relation to parametric templates generated from a control cohort.

In conclusion, ASL MR imaging enables contrast-material-free and simultaneous measurement of CBF, ATT, and flow territory. Our data suggest that ATT and flow territory may be better predictors of high-grade unilateral ICA stenosis (≥70%) than CBF in asymptomatic patients diagnosed by carotid ultrasonography. Combination of CBF/ATT/flow territory may be of clinical interest for providing more comprehensive assessment of hemodynamics and indication to treatment.
